# Focal adhesions control cleavage furrow shape and spindle tilt during mitosis

**DOI:** 10.1038/srep29846

**Published:** 2016-07-19

**Authors:** Nilay Taneja, Aidan M. Fenix, Lindsay Rathbun, Bryan A. Millis, Matthew J. Tyska, Heidi Hehnly, Dylan T. Burnette

**Affiliations:** 1 Department of Cell and Developmental Biology, Vanderbilt University School of Medicine, Nashville, TN 37232, USA; 2 Department of Cell and Developmental Biology, SUNY Upstate Medical University, Syracuse, NY 13210, USA

## Abstract

The geometry of the cleavage furrow during mitosis is often asymmetric *in vivo* and plays a critical role in stem cell differentiation and the relative positioning of daughter cells during development. Early observations of adhesive cell lines revealed asymmetry in the shape of the cleavage furrow, where the bottom (i.e., substrate attached side) of the cleavage furrow ingressed less than the top (i.e., unattached side). This data suggested substrate attachment could be regulating furrow ingression. Here we report a population of mitotic focal adhesions (FAs) controls the symmetry of the cleavage furrow. In single HeLa cells, stronger adhesion to the substrate directed less ingression from the bottom of the cell through a pathway including paxillin, focal adhesion kinase (FAK) and vinculin. Cell-cell contacts also direct ingression of the cleavage furrow in coordination with FAs in epithelial cells—MDCK—within monolayers and polarized cysts. In addition, mitotic FAs established 3D orientation of the mitotic spindle and the relative positioning of mother and daughter centrosomes. Therefore, our data reveals mitotic FAs as a key link between mitotic cell shape and spindle orientation, and may have important implications in our understanding stem cell homeostasis and tumorigenesis.

## Results

**Figure 1:**
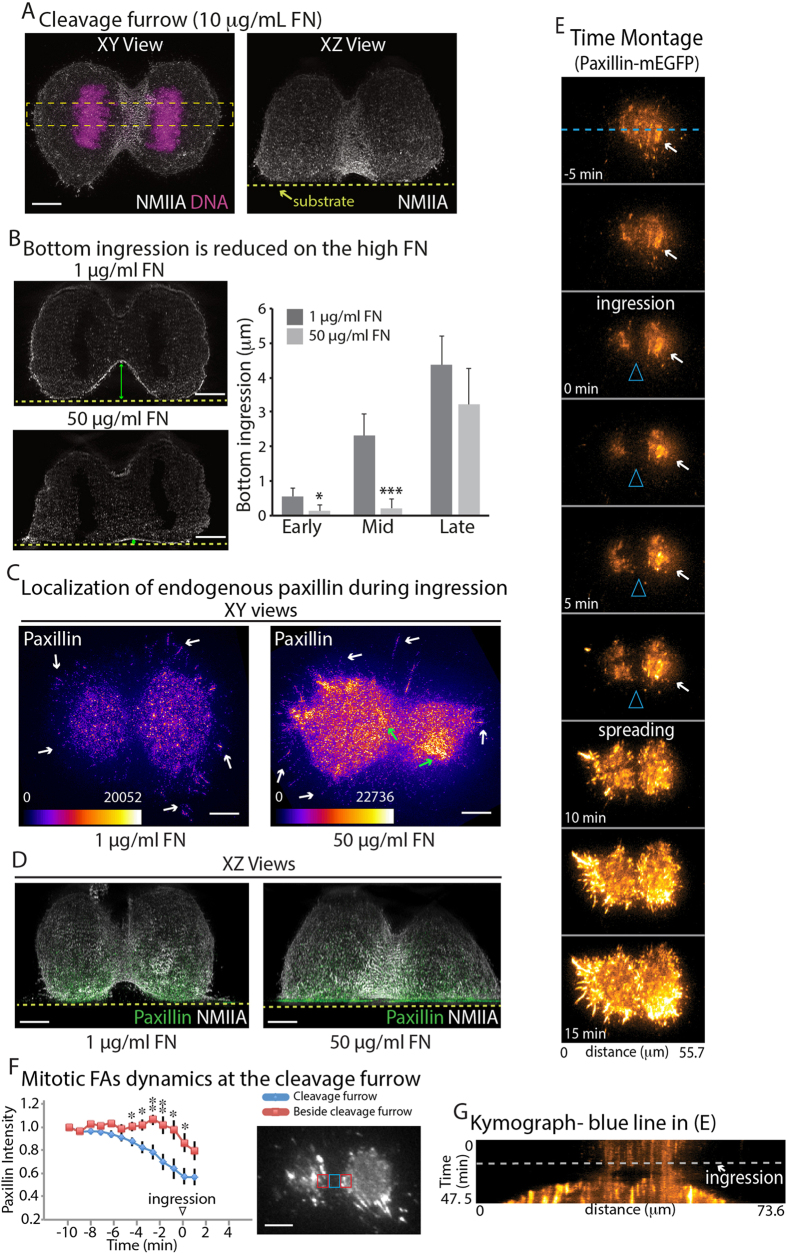
Substrate adhesion controls the symmetry of the cleavage furrow. (**A**) XY and XZ views of the cleavage furrow of a HeLa cell cultured on 10 μg/mL FN and stained for endogenous NMIIA and DNA. (**B**) XZ views of the cleavage furrow of cells cultured on low (1 μg/mL) and high (50 μg/mL) FN substrates. XZ projections were made from a similar sized ROI as in (**A**). Ingression from the bottom (double headed green arrow) was measured as the distance between the substrate (dotted yellow line) and the bottom of the cleavage furrow. Cells were grouped based on the height of the cleavage furrow into early (>15 μm), mid (9–15 μm) and late (3–9 μm) stages of ingression. Measurements were made on 34 cells and 42 cells for 1 μg/mL and 50 μg/mL FN, respectively, across 6 independent experiments for each condition (see Methods). (**C**) XY views of HeLa cells at anaphase stained for paxillin, cultured on low and high adhesive substrates. Look up table is fire and color bars show the gray scale values of the images. White arrows show retraction fiber adhesions and green arrows show mitotic FAs. (**D**) Merged XZ views of HeLa cells at anaphase stained for paxillin (green) and NMIIA (gray) cultured on low and high adhesive substrates. XY views are shown in Figure S1C. (**E**) TIRF time montage of a HeLa cell expressing Paxillin-mEGFP and H2B-mCherry cultured on high adhesive substrate undergoing anaphase imaged using TIRF microscopy. Ingression starts at 0 min and the arrowheads indicate the position of the cleavage furrow. Arrows denote the side with larger adhesions maintained until the daughter cells start spreading at 10 min. (**F**) Quantification of relative paxillin intensity comparing adhesions underneath the cleavage furrow (red ROI in inset) and immediately adjacent to the cleavage furrow (blue ROI in inset). Measurements were made from 7 cells across 5 independent experiments. (**G**) Kymograph created from blue line in (**C**). Dotted line denotes the onset of ingression. * denotes p < 0.05 and ** denotes p < 0.01; Scale bars, 5 μm. Error bars show standard error of the mean (SEM).

We started our exploration of how adhesions shape the cleavage furrow using a classic model of mitosis: single cells dividing in culture. FAs are formed through binding of specific integrins to extracellular matrix (ECM) proteins. Therefore, we plated HeLa cells on coverslips coated with 10 μg/mL fibronectin (FN) (Fig. 1A) as previously used for studies of cell migratio^1^. After fixation, DNA was labeled with Hoechst and myosin IIA was labeled with fluorescent antibodies. Hoechst allowed us to identify cells in mitosis and myosin IIA labeling allowed us to visualize cell shape. 3D structured illumination microscopy (SIM)^2^,^3^ of cells in anaphase B/telophase revealed the cleavage furrow was symmetrical in the XY plane, which indicated the cell had ingressed equally from either side (Fig. 1A). However, XZ projections revealed the cleavage furrow often ingressed further from the top of the cell than the bottom (Fig. 1A), consistent with previous findings using adhesive NRK cells^4^. We next wanted to test if the geometry of the cleavage furrow was dependent on the extent of adhesion to the substrate.

Studies during interphase reported cells make smaller and less stable FAs on coverslips coated with low densities of FN (i.e., <5 μg/mL) and larger and more stable FAs on substrates coated with high concentrations of FN (>30 μg/mL)^5^. We predicted increasing adhesions with a “high” FN substrate would result in less ingression from the bottom of the cell and, thus, an asymmetrical cleavage furrow. Therefore, we plated cells on low (1 μg/mL FN) and high (50 μg/mL FN) adhesive substrates and then analyzed cell shape. Cells were grouped into three stages of anaphase (i.e., early, mid, and late) based on the axial diameter of the contractile ring (see Figure S1 and Methods). SIM allowed us to note for the first time a ~4-fold and ~13-fold increase in ingression from the bottom on the low adhesive substrate compared to the high adhesive substrate during early and mid-anaphase, respectively (Fig. 1B). Notably, high bottom ingression was measured during late anaphase on both low and high FN. Midbody formation was observed at varying distances from the substrate, suggesting other mechanisms could be operating during the final stages of cytokinesis. Furthermore, using this microscopic approach, we also calculated the aspect ratio of the contractile ring to test whether these resistive forces were transmitted to the ring. The contractile ring was significantly more circular on the low adhesive substrate (1 μg/mL FN) (Figure S1B), while on the high adhesive substrate (50 μg/mL FN) the ring was flatter and had a significantly increased aspect ratio (Figure S1C, 1.3 compared to 1). Together, these initial observations suggest increasing adhesion to the substrate drives asymmetrical ring contraction by preventing ingression from the bottom of the cell.

We next wanted to understand the nature of the adhesive forces that control 3D shape of the cleavage furrow. A previously described mitotic population of adhesions can occur within retraction fibers^6^,^7^. Retraction fibers form during mitotic entry and are thought to contribute to the XY orientation of the mitotic spindle during metaphase and further dictate the pattern of cell spreading after mitotic exit of the two daughter cells^8^,^9^. Each retraction fiber has a FA mediating attachment to the substrate. We hypothesized retraction fibers on high FN substrates would form larger/more stable FAs and these may be driving asymmetric furrow ingression. To test this hypothesis, we localized the FA protein, paxillin, in cells during anaphase. Surprisingly, we found no difference in the sizes of FAs at the ends of retraction fibers (Fig. 1C, white arrows). However, we did observe an additional population of adhesions distinct from retraction fibers, located directly underneath the cell body (Fig. 1C, green arrows and Fig. 1D). Consistent with reported data for interphase cells, mitotic cells assembled more adhesions and had a greater spread area on the high FN substrate compared to the low FN samples (Fig. 1C, S2C-D).

To understand the temporal dynamics of mitotic adhesions, we turned to live cell TIRF microscopy. HeLa cells expressing EGFP-Paxillin and H2B-mCherry were imaged starting at metaphase through anaphase and telophase. Small, but distinct and highly dynamic adhesions were observed underneath the cleavage furrow, undergoing continuous formation and turnover (Fig. 1E, Movie S1). Mitotic FAs underneath the furrow disappeared just prior to cleavage furrow ingression (Fig. 1E,F). This was followed by initiation of cell spreading (Fig. 1E–G). Therefore, mitotic FAs exist during the metaphase to anaphase transition and are spatially and temporally distinct from retraction fibers.

**Figure 2:**
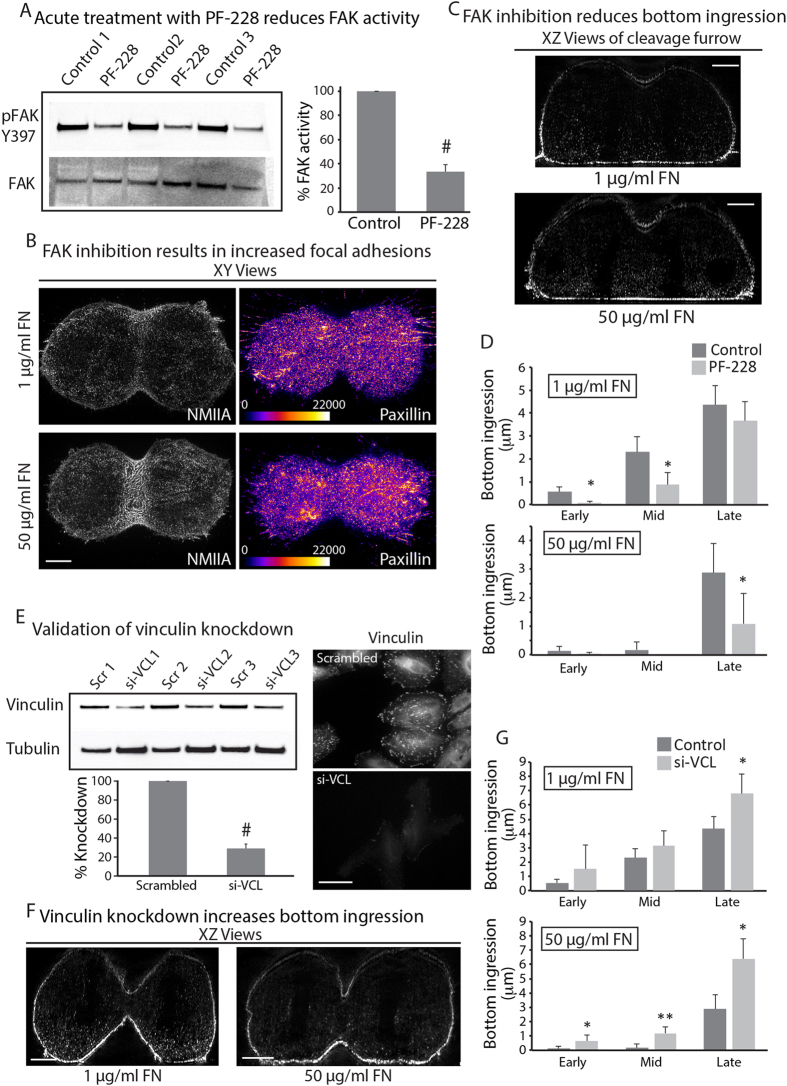
Molecular mechanisms governing mitotic Fas. (**A**) Western blot to validate acute inhibition of FAK using the FAK inhibitor (PF-228). Lysates were prepared from cells treated for 10 minutes versus untreated controls from 3 independent experiments. Total FAK was used as loading control. Intensities for each lane were normalized to the respective loading controls. (**B,C**) XY (**B**) and XZ (**C**) views of HeLa cells at anaphase stained for NMIIA and paxillin, cultured on low (top) and high (bottom) adhesive substrate treated with 1 μM FAK inhibitor PF-228. (**D**) Quantification of bottom ingression comparing FAK inhibitor treated versus untreated control cells cultured on low and high FN substrates. Cells were grouped into early, mid and late anaphase based on the height of the cytokinetic ring as in Fig. 1. Measurements were made on 31 cells across 4 independent experiments and 30 cells across 3 independent experiments for 1 and 50 μg/mL FN, respectively. (**E**) Validation of si-RNA knockdown of vinculin using western blotting and immunofluorescence. For western blotting, intensities for each lane were normalized to the respective tubulin loading controls. Each knockdown was then normalized to the respective scrambled (Scr) control. N = 3 independent experiments, each performed with pooled siRNAs containing 4 independent siRNAs. For immunofluorescence, control and knockdown HeLa cells were stained for endogenous vinculin. (**F**) XZ views of vinculin knockdown HeLa cells at anaphase cultured on low and high adhesive substrates **G**) Quantification of bottom ingression comparing vinculin knockdown versus control cells cultured on low and high adhesive substrates. Cells were grouped into early, mid and late anaphase as in Fig. 1. Measurements were made on 17 cells across 3 independent experiments and 24 cells across 4 independent experiments for 1 μg/ml and 50 μg/ml FN, respectively. Each experiment was performed using pooled siRNAs containing 4 independent siRNAs. Scale bars in (**B,C**) and (**F**), 5 μm; (**E**), 100 μm. *denotes p < 0.05 and ** denotes p < 0.01. Error bars in (**A**,**D**,**E**,**G**) show standard error of the mean (SEM).

We next wanted to investigate the molecular mechanisms controlling the function of mitotic FAs. We hypothesized similar mechanisms control adhesion dynamics during interphase and mitosis. Thus, we targeted molecular players known to regulate adhesion strength during interphase. Inhibition of focal adhesion kinase (FAK) has been reported to stabilize FAs by preventing their disassembly^10^. Thus, creating stable FAs through FAK inhibition during mitosis should further reduce ingression from the bottom. Therefore, we inhibited FAK with an acute treatment of a specific drug, PF-228^11^. To prevent adhesion disassembly only during mitosis, we treated cells with 1 μM PF-228 for 10 minutes before fixation and immuno-labeling so that any cells analyzed during anaphase would have been targeted during metaphase or later. Drug treatment reduced FAK auto-phosphorylation by 66.7+/−9.5% (Fig. 2A) and resulted in larger mitotic adhesions in cells on low FN (Fig. 2B compared with Fig. 1C, Figure S2C) (Fig. 2C,D). Bottom ingression on high FN was also reduced upon PF-228 treatment compared to control cells. There was also a ~8-fold decrease in bottom ingression during early anaphase and greater than 2-fold reduction during mid anaphase when cells were on low FN (Fig. 2C,D). Thus, FAK inhibition can enhance asymmetric ingression even when cells are on a low adhesive substrate (Fig. 2C,D).

Vinculin is part of the molecular clutch that links adhesions to the actin cytoskeleton^11^. To determine if vinculin also serves as a clutch to regulate cleavage furrow ingression, we used siRNA to knockdown endogenous vinculin protein (Fig. 2E). Indeed, we observed knockdown of vinculin caused a nearly 10-fold increase in ingression from the bottom on the high adhesive substrate (Fig. 2F–G). Taken together, these findings indicate FAK and vinculin regulate cleavage furrow ingression.

**Figure 3:**
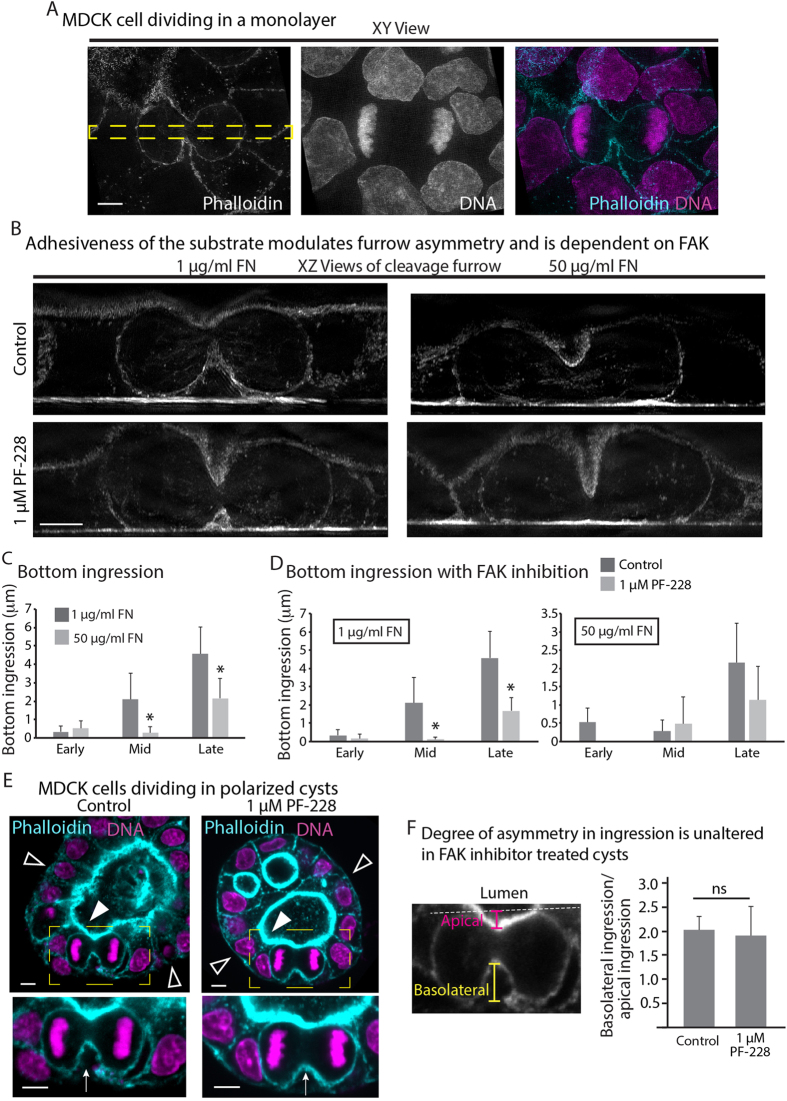
Adhesiveness of the substrate also controls the shape of the cleavage furrow in epithelial systems. (**A**) XY view of a cell dividing in a MDCK monolayer (**B**) XZ views of control (top row) and FAK inhibitor treated (bottom row) cells dividing within a monolayer grown on low (left column) and high (right column) adhesive substrates. The XZ projections of the cleavage furrow were created from a thin slice (marked by dotted yellow box in (**A**)) passing through the long axis of the cell. (**C**) Quantification of basolateral ingression comparing low and high adhesive substrates. (**D**) Quantification of basolateral ingression comparing control and treated cells on low and high adhesive substrates. Cells were grouped into early (>10 μm), mid (6–10 μm) and late (1–5 μm) anaphase based upon the height of the cytokinetic ring. For the graph comparing control cells, measurements were made on 33 cells across 3 independent experiments and 36 cells across 4 independent experiments for 1 μg/ml and 50 μg/ml FN respectively. For the graph comparing FAK treated cells with control cells, measurements were made on 23 cells for 1 μg/ml FN and 24 cells for 50 μg/ml FN across 3 independent experiments for each condition. (**E**) Cross sections through a MDCK cyst showing cells at anaphase in either control (left) or FAK inhibitor treated (right) cysts stained with phalloidin (cyan) and DAPI (magenta). Shown are maximum projections of six 200 nm Z slices, with magnified views of the region marked by the dotted yellow rectangle below. (**F**) Quantification of degree of asymmetry in furrow ingression comparing control and FAK inhibitor treated cysts. Y axis represents the degree of asymmetry, calculated as ratio of basolateral versus apical ingression as shown in the inset. Measurements were made on 11 control cysts across 5 independent experiments and 4 PF-228 treated cysts across 3 independent experiments. Scale bars: 5 μm; * denotes p<0.05.

Although single isolated cells are a powerful model for exploring mechanisms that govern the attachment of mitotic FAs to the ECM, cells are often also attached to other cells *in vivo*. This is particularly true for cells within an epithelial sheet. Epithelial cells form FAs on their basal domain and tight junctions and adherens junctions (i.e., cell-cell contacts) on their apical and lateral domains, respectively^12^,^13^. Of note, cleavage furrows of epithelial cells dividing *in vivo* tend to ingress from the basolateral side towards the apical side^14^,^15^,^16^,^17^,^18^, and this type of asymmetric ingression is required for proper differentiation of neural epithelia^15^. We hypothesized a low adhesive state on the bottom (i.e., basolateral side) of epithelial cells could allow the cleavage furrow to detach and pull itself towards cell-cell contacts (i.e., apical). To test this hypothesis, we grew monolayers of MDCK cells on low FN, and found the cleavage furrow did ingress from the bottom (Fig. 3A, top panels in Fig. 3B). Cells on high FN showed ~8 fold less ingression from the bottom and more from the top compared to low FN during mid-anaphase (Fig. 3B,C). This data suggested ECM adhesion determines the shape of the cleavage furrow in mitotic cells that are integrated into epithelial monolayers.

To determine if mitotic adhesions shape the cleavage furrow of epithelial cells in a regulated way, we inhibited FAK and measured furrow ingression. FAK inhibition resulted in a dramatic 16-fold decrease in furrow ingression from the bottom of MDCK cells on the low FN during mid-anaphase, as well as small reduction on high FN during early and late anaphase (Fig. 3B,D). To further test if FAK inhibition was working through mitotic adhesions to the ECM, we analyzed ingression in cells lacking ECM attachment due to presence of a very soft matrix. For these experiments, we grew MDCK cells in polarized 3D cysts sitting on coverslips^19^. The basolateral side of the cells was on the outside of the cysts (Fig. 3E, open arrowheads) and the apical side was on the luminal side (Fig. 3E, closed arrowheads). In the case of cells at the sides of the cyst that have no attachment to the coverslip in contact with the liquid medium, the cleavage furrow of these cells ingressed completely towards the apical (luminal) side of the cell with or without FAK inhibition (Fig. 3E (arrow) and 3F). Thus, the influence of FAK on furrow shape requires adhesions to the ECM. To visualize whether epithelial cells *in vivo* also ingress their cleavage furrow from the basolateral domain, we visualized furrow ingression in cells dividing near the base of the crypt of mouse small intestine. We observed that furrow ingression indeed proceeds from the basolateral domain (Figure S3), suggesting that the base of the crypt behaves like a low adhesive environment.

**Figure 4:**
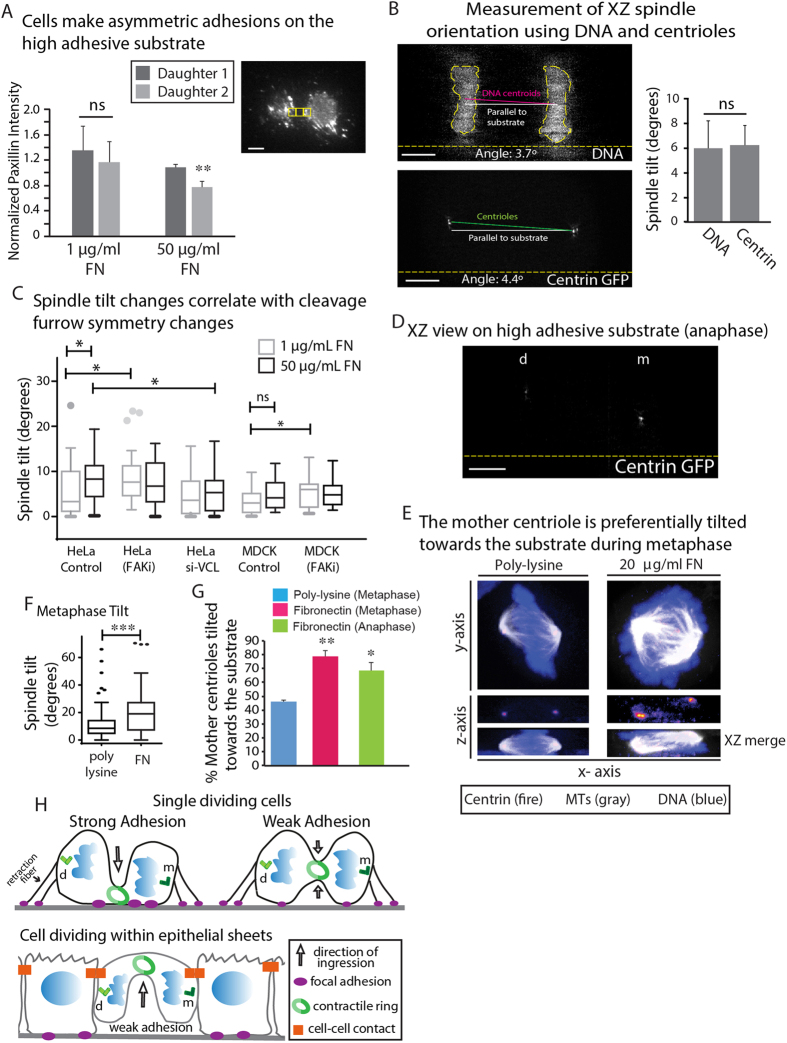
Adhesiveness of the substrate modulates the XZ orientation of the spindle. (**A**) Quantification of degree of asymmetry in attachment on low and high adhesive substrates using TIRF. ROIs on either side of the cleavage furrow ROI were compared. Measurements were made from 8 cells across 5 independent experiments as in Fig. 2D for 50 μg/mL FN and 4 cells across 4 independent experiments on 1 μg/mL FN. (**B**) Methods used to quantify spindle tilt. XZ tilt of the spindle was calculated by either measuring the angle between the line joining the centroids of the chromosomes (solid magenta line) and the substrate (solid white line) or by measuring the angle between the line joining the centrosomes (solid green line) and the substrate (solid white line) using a HeLa cell line stably expressing GFP centrin. Dotted yellow line shows the substrate. Graph shows comparison of the two methods. Spindle tilt was measured using both methods in 28 cells across 5 independent experiments. (**C**) Tukey plots comparing spindle tilt measured using the DNA centroids method across all experimental conditions tested for HeLa and MDCK cells in the study (**D**) XZ view of a HeLa cell stably expressing centrin GFP at anaphase on a high adhesive substrate showing the mother (m) and daughter (d) centrosomes. (**E**) Shown are confocal images of cells in metaphase on FN or poly-L-lysine, with XY and XZ views of DNA (blue), centrin (fire) and tubulin (gray). (**F**) Tukey plots comparing the average spindle tilt on poly-L-lysine versus FN during metaphase. (**G**) Graph comparing the propensity of the mother centrosome being tilted towards the substrate during metaphase and anaphase. H) Our model for how mitotic adhesions control the 3D shape of the cleavage furrow of single cells and cells within epithelial monolayers. m and d indicate mother and daughter centrosomes, respectively. Scale bars, 5 μm. * denotes p < 0.05, ** denotes p < 0.01 and *** denotes p < 0.005. Error bars in (**A**,**B**,**G**) shows standard error of the mean (SEM).

We next wanted to explore what other cellular processes could be affected by mitotic FAs. Interestingly, during anaphase we noted one daughter was shorter than the other on high FN (Fig. 1D). Corresponding XY views indicated the shorter cell had larger FAs (Figure S1). Indeed, quantification of FAs on either side of the cleavage furrow from our live cell TIRF data confirmed this asymmetric attachment occurs only on the high FN substrate (Fig. 4A). The observed cellular shape during anaphase (Fig. 1D) suggested one pole of the mitotic spindle could be closer to the substrate than the other, characteristic of a tilted spindle in XZ. Therefore, we took two approaches to determine the tilt of an anaphase mitotic spindle by measuring the angle between the substrate and a line joining the centroids of the DNA or the spindle poles marked with centrin (Fig. 4B). Each method yielded statistically identical spindle angles (Fig. 4B). Using these two methods, we noted that on high adhesive substrate (50 μg/mL FN) anaphase spindles demonstrated a mean angle of 9.7°+/−3.6°, whereas on low adhesive substrate a mean angle of 5.5+/−2.4° was calculated (1 μg/mL FN) (Fig. 4C,D). To test whether changes in the shape of the cleavage furrow also affect spindle tilt, we measured spindle tilt upon inhibition of FAK and knockdown of vinculin, which increases and decreases the strength of adhesion, respectively (as noted in Fig. 2). Upon inhibition of FAK, spindle tilt was significantly increased on a low adhesive substrate. Conversely, with vinculin depletion it was decreased on the high adhesive substrate. In MDCK monolayers a spindle angle of 0–5° was calculated on either low or high FN. However, upon addition of the FAK inhibitor a mean spindle angle of 5.5° was observed for cells grown on 1 μg/ml FN compared to a mean angle of 3° for control MDCK cells. Thus in both MDCK and HeLa cells, increasing adhesion by inhibiting FAK causes an increase in spindle tilt.

Of note, we found using a Centrin-GFP cell line that the mother (i.e., older) spindle pole, marked by the spindle pole with a brighter centrin signal^20^ (Fig. 4D), was preferentially tilted towards the substrate. We confirmed these results using an alternative approach, where we used an antibody to cenexin, which specifically marks the mother centrosome^21^,^22^,^23^, and observed similar results (Figure S4). In addition, we noted that metaphase cells on FN demonstrated a mean spindle angle of 20° towards the substrate (Fig. 4E–G). Thus, spindle tilt precedes furrow ingression, suggesting the two processes are co-regulated but not mechanically coupled, (i.e. the cleavage furrow is not tilting the spindle by physically pushing on it).

## Discussion

A balance between contraction and adhesion to the substrate determines the three dimensional shape of cells during interphase^24^. Our results are highly suggestive of a similar balance existing between contractile forces generated by the ring and adhesive resistive forces generated by cell adhesion to the substrate during mitosis (Fig. 4H). In single cells, this model suggests that strong adhesion to the substrate would create high resistance to the contractile force generated by the ring, in turn resulting in asymmetric ingression from the top of the cell. Under low adhesion conditions, there would be less resistive force and therefore the top and bottom of the cell ingress symmetrically (Fig. 4H). We also show cell-cell contacts in epithelial cells directing the ingression of the cleavage furrow. The resultant shape of the cleavage furrow under low adhesion to the substrate is similar to that usually observed *in vivo*, such as in *Drosophila* embryos^16^, mouse neuroepithelium^15^ and intestinal crypts^17^ (Figure S3).

Based on our data, we attribute the resistive force largely to the mitotic FAs underneath the cell body. First, they are the largest FAs prominent throughout mitosis^25^. Second, we show that mitotic FAs are responsive to adhesiveness of the substrate (Fig. 1) and are regulated by FAK and vinculin activity (Fig. 2). Interestingly, we did not observe significant changes in retraction fiber density in response to either substrate adhesiveness or signaling. So what is the role of retraction fibers? Previous work using patterned substrates showed the position of the cleavage furrow and XY orientation of the mitotic spindle is determined by adhesive patterns^8^. Specifically, the spindle aligns with the axis of strongest external force in XY exerted by retraction fibers or cell-cell contacts^26^,^27^. Furthermore, retraction fibers also maintain the orientation of the spindle parallel to the substrate^28^. It has recently been reported force generated by retraction fibers activates β-1 integrin on the mid lateral cortex^25^. Therefore, the ultimate orientation of the spindle in three dimensions is likely determined by an interplay of retraction fibers maintaining the spindle parallel to the substrate and mitotic FAs pulling the mother centrosome towards the substrate through a yet to be defined mechanism.

The XZ orientation of the spindle and specific placement of the oldest mitotic centrosome have been implicated in the stereotyped behavior of Germline Stem Cells (GSCs) and neuroblasts in Drosophila^29^,^30^ and in several invertebrate cell types^22^,^29^,^30^,^31^,^32^,^33^,^34^. We observed the mother spindle pole preferentially localizes to the daughter cell with more mitotic FAs. This observation may explain the original observation by Yamashita *et al*. where the oldest mitotic centrosome remains closer to the niche in dividing germline stem cells (GSCs)^30^, In addition to GSCs, mammalian gut stem cells orient their divisions so that the cell that retains contact with the ECM retains stemness^35^. Therefore, our findings suggest mitotic FAs may play multiple roles in stem cell homeostasis, including anchoring stem cells to a niche and retention of specific stem cell factors such as recruitment of the mother spindle pole^34^. Deregulation of normal stem cell differentiation has been implicated to cause tumorigenesis^36^. Therefore, the balance of adhesive forces determining the position and ultimate fate of dividing stem cells could also play a role during tumorigenesis.

## Materials and Methods

### Cell culture and chemicals

HeLa (ATCC, CCL-2) and HeLa centrin-EGFP^20^ cells were cultured in growth media comprised of DMEM (Mediatech, Inc., Manassas, VA, 10-013-CV) containing 4.5 g/L L-glutamine, L-glucose, sodium pyruvate and supplemented with 10% fetal bovine serum (Sigma-Aldrich, St. Louis, MO, F2442). MDCK II cells were generously provided by Dr. Ian Macara. Growth substrates were prepared by coating #1.5 glass coverslips (*In Vitro* Scientific, D35C4-20-1.5N or D35-20-1.5N) with 1,10 or 50 μg/mL FN (354008, Corning) in PBS (Mediatech, Inc., 46-013-CM) at 37 °C for 1 hour. Cells were plated on a growth substrate and then experiments were performed the next day. For protein expression, cells were transiently transfected using Fugene 6 (Promega, Madison, WI, E2691) according to the manufacturer’s instructions overnight in a 25-cm^2^ cell culture flask (Genessee Scientific Corporation, San Diego, CA, 25-207) before plating on a growth substrate. Culturing of polarized MDCK cysts was done as described previously^19^. Briefly, MDCK cells were sparsely seeded as single cells on a growth substrate coated with 5 μg/mL laminin and overlaid with 5% reduced growth factor Matrigel (Corning). Polarized cysts were obtained after about a week of incubation, with Matrigel replenished every day.

The FAK inhibitor PF-228 (PZ0117) was purchased from Sigma. Alexa Fluor 488-phalloidin (A12379), Alexa Fluor 488-goat anti-mouse (A11029), Alexa Fluor 488-goat anti-rabbit (A11034), Alexa Fluor 568-goat-anti-rabbit (A11011), Alexa Fluor 568-goat anti-mouse (A11004), Alexa Fluor 647-goat-anti-mouse (A21235), anti-rabbit HRP (65–6120) and anti-mouse HRP (62–6520) antibodies were purchased from Life Technologies (Grand Island, NY). Rabbit-anti-Myosin IIA (909801) was purchased from BioLegends (San Diego, CA). Mouse anti-Paxillin (810051) was purchased from BD Biosciences. Rabbit anti-pFAK Y357 (ab81298) was purchased from Abcam. Mouse anti-FAK (BD Biosciences) was a generous gift from the lab of Dr. Irina Kaverina. Paraformaldehyde (15710) was purchased from Electron Microscopy Sciences (Hatfield, PA). Triton X-100 (BP151100) was purchased from Fischer Scientific (Suwanee, GA).

### Plasmids

EGFP-Paxillin-KM-N-10 was a gift from Michael Davidson (Addgene plasmid # 56283).

### Drug treatments

Treatment with FAK inhibitor PF-228 was done as described previously^12^. Briefly, the drug was diluted to a final concentration of 1 μM in serum free DMEM pre-heated to 37 °C and applied for 10 minutes. Following drug treatment, the drug was washed out and cells were fixed for staining. For western blotting, cells were washed with PBS and lysed using Cell Lytic M (Sigma) reagent containing 1X Protease Inhibitor Cocktail (Sigma), 1X Phosphatase Inhibitor Cocktail II (Sigma) and 1 μM PF-228 using scraping.

### Knockdown of vinculin using si-RNA

Smart Pool Accell siRNAs against vinculin (E-009288-00-0005) and scrambled control (D-001910-10-05) were purchased from GE Dharmacon. Knockdown experiments were performed in 24 well plates using Lipofectamine 2000 (1690146, Life Technologies) according to the instructions provided by the manufacturer. Knockdown was performed for 72 hours, following which cells were plated on the growth substrate. For western blotting, cells were washed with PBS and lysed using Cell Lytic M reagent (Sigma) containing 1X Protease Inhibitor Cocktail.

### Tissue Processing and Staining

Duodenum from the small intestine of 2 month old C57/BL6 mice was harvested and immediately fixed and sub-dissected in 4% paraformaldehyde in PBS for 30 min. Tissue was then washed in PBS and cryoprotected overnight in 30% sucrose at 4 °C before embedding in OCT and freezing over an acetone-dry ice bath. Frozen blocks were stored at −80 °C until the time of sectioning. OCT blocks were sectioned using a Leica CM 1950 cryostat, at a thickness of 10 μm and melted on *Superfrost Plus* microscope slides (Fisher Scientific). All slides were kept at −80 °C until the time of staining. Slides were thawed and OCT washed out in room temperature PBS three times for 5 min each before staining with AlexaFlour488-conjugated Phalloidin (1:200, Molecular Probes) and DRAQ5 (1:200, ThermoScientific) for 2 hours. Subsequent to washing in PBS, samples were mounted with ProLong Gold antifade reagent (Molecular Probes) and #1.5 coverslip (Electron Microscopy Sciences).

### Metaphase imaging

Cells were seeded on glass coverslips (#1.5, Warner Instruments) and grown to sub-confluence for immunofluorescence confocal microscopy. Cells were then fixed (cold methanol) and stained as previously described^37^. Images were taken on a Perkin Elmer spinning disk confocal microscope: Nikon Eclipse Ti microscope, Plan-Apochromat 100x/1.4 Oil DIC objective and a Hamamatsu C9100-50 EMCCD camera. The entire cell was imaged at 0.2-μm step-intervals and displayed as maximum projections (ImageJ). The fluorescence range of intensity was adjusted identically for each image series. Orthogonal images of mitotic spindle were processed with ImageJ software. For measuring spindle angles in 2-dimensional cultures, anti-centrin or centrin-GFP was used to indicate spindle pole positions, and spindle angle measurements were carried out as previous described^37^.

### Confocal Microscopy and Image Processing

Confocal imaging of tissue sections was accomplished using a Nikon TiE inverted microscope outfitted with an A1R-plus point-scanning confocal, 100× 1.49NA Apo TIRF objective, and piezo stage (Nikon Instruments, Inc.). Subsequent to image stack acquisition, datasets were deconvolved offline using Richardson-Lucy 3D deconvolution. Both image acquisition as well as processing, including deconvolution, was accomplished through the use of NIS-Elements software (Nikon Instruments, Inc.).

### Structured Illumination Microscopy

SIM imaging and processing was performed on a GE Healthcare DeltaVision OMX equipped with a 60 × 1.42 NA Oil objective and sCMOS camera.

### Live Cell TIRF microscopy

Live cell TIRF imaging was performed on a Nikon N-STORM microscope using a Nikon 100x Plan Apo 1.49 NA objective and an Andor iXon Ultra EMCCD camera. Cells in metaphase were identified using the H2B mCherry channel, following which images were acquired every 10–30 s in TIRF in the green channel. Cells were imaged until they had completed telophase and initiated cell spreading. A camera gain of 110 was used. The hardware was controlled using Nikon Elements AR software.

### Western Blotting

Gel samples were prepared by mixing cell lysates with LDS sample buffer (NP0007, Life Technologies) and Sample Reducing Buffer (NP0009, Life Technologies) and boiled at 95 °C for 5 minutes. Samples were resolved on Bolt 4–12% gradient Bis-Tris gels (NW04120BOX, Life Technologies). Protein bands were blotted onto a nylon membrane (Millipore). Blots were blocked using 5% NFDM (33368, Research Products International Corp, Mt. Prospect, IL) in TBST. Antibody incubations were also performed in 5% NFDM in TBST. Blots were developed using Immobilon Chemiluminescence Kit (WBKLS0500, Millipore).

### Data Quantification

All image processing was performed on ImageJ. For measuring relative bottom ingression, images were first rotated so that cells were aligned with their long axis parallel to the substrate. 3D projections were then made along the X-axis a thin slice ROI passing through the center of the cell. Relative bottom ingression was measured as the distance from the substrate to the bottom of the cleavage furrow. For measuring aspect ratio of the ring, the cell was rotated 90 degrees so that its long axis was perpendicular to the substrate and an X-projection was created using a thin slice ROI passing through the contractile ring. Ring aspect ratio was measured as the ratio between the horizontal and axial diameter of the contractile ring. Cells were then divided into three stages of anaphase according to the height of the contractile ring. 3–9 μm for late, 9–15 μm for mid and >15 μm for early anaphase for HeLa cells; for MDCK cells, 1–5 μm for late, 5–10 μm for mid and <10 μm for late anaphase. For measurement of degree of asymmetry in cyts, Z slices were maximum projected and two lines parallel to the long axis of the cell were drawn to mark the highest and lowest luminal and the basal planes, respectively. Apical and basolateral ingression was measured as distance between the furrow and the apical or basal plane. Cells across multiple independent experiments were pooled and statistical analyses were performed using Student’s T-Test.

For quantification of TIRF data, four ROIs were drawn: one at the cleavage furrow and two on either side of the cleavage furrow, and one in the background. The largest size ROI that could fit all cleavage furrows in the data set was empirically set, and identical sized ROIs were placed on either side of this central ROI. We used identical sized ROIs for all measurements and they were simply placed by keeping the furrow ROI at the center of the two daughter cells blindly to reduce any bias. All intensities were first background corrected using the average background mean intensity. The ingression of the cleavage furrow was designated as T = 0 min and frames from 10 minutes before and after furrow ingression at 1 minute intervals were used for analysis. Each intensity value was normalized to T = −10 min to account for variation in intensity values due to expression levels. Due to asymmetry in attachment of the daughter cells, the cleavage furrow ROI was compared to the brighter adjacent ROI (designated Side 1 in the excel sheet). For quantification of degree of asymmetry in the attachment of cells, we normalized the intensity values measured from the ROI in the less attached cell to T = −10 min of the brighter ROI (designated Side 2 in the excel sheet). The average normalized intensities were then compared using Mann Whitney U Test, since these data were not assumed to be uniformly distributed.

For quantification of adhesion density, the bottom ten Z-stacks were maximum projected and integrated intensity per unit area was calculated using ImageJ software. For quantification of spindle tilt, two methods were employed. In the first method, we measured tilt as the angle between the line joining the centroids of the DNA intensity and the substrate. In the second method, measured tilt as the angle between the substrate and the line joining the centrioles, imaged using a HeLa cell line stably expressing GFP tagged centrin protein. Statistical comparison of these methods showed that they yielded nearly the same value, and we use the DNA method for the remainder of the study.

Graphs were created using Microsoft Excel and GraphPad Prism.

## Additional Information

**How to cite this article**: Taneja, N. *et al*. Focal adhesions control cleavage furrow shape and spindle tilt during mitosis. *Sci. Rep*. **6**, 29846; doi: 10.1038/srep29846 (2016).

## Supplementary Material

Supplementary Information

Supplementary Movie S1
