# Supplementary material for: Focal adhesions control cleavage furrow shape and spindle tilt during mitosis
Source: Sci Rep. 2016 Jul 19;6:29846. doi: 10.1038/srep29846 (PMC4949487; doi:10.1038/srep29846)

# Focal adhesions control cleavage furrow shape and spindle tilt during mitosis

Nilay Taneja, Aidan Fenix, Lindsay Rathbun, Bryan Millis, Matthew Tyska, Heidi Hehnly and Dylan Burnette*

## Supplementary Information

## Supplemental Figure Legends

**Figure S1- Substrate adhesions control the symmetry of the cleavage furrow**

A) XZ views of the contractile ring (pseudo-colored yellow) with respect to the cell body (gray) comparing 1 µg/mL and 50 µg/mL FN. Images were aligned perpendicular to the long axis of the cell and XZ views were created using a similar sized ROI as in Figure 1A. B) Quantification of aspect ratio of the ring comparing 1 and 50 µg/mL FN. The aspect ratio was calculated as the ratio of the horizontal to the axial diameter of the ring. Measurements were made on 34 cells and 42 cells for 1 µg/mL and 50 µg/mL FN, respectively, across 6 independent experiments for each condition C) XY views of HeLa cells at anaphase on low and high adhesive substrates stained for endogenous paxillin (green) and NMIIA (gray). * denotes $p<0.05$; Scale bars, 5 µm. Error bars show standard error of the mean (SEM).

**Figure S2- Validation of inhibition of FAK using PF-228 and knockdown of vinculin using siRNA**

A) Full western blots representing three independent experiments to validate the inhibition of FAK activity using an acute 10-minute treatment with FAK inhibitor PF-228. B) Full western blots representing three independent experiments to validate the knockdown of vinculin using siRNA. Asterisk indicates the residual anti-vinculin antibody not removed during the stripping step. C) Quantification of paxillin intensity normalized to the spread area for control and PF-228 treated cells. D) Quantification of spread area

during anaphase for control and PF-228 treated cells. Measurements were made on 17 cells across 3 independent experiments, 19 cells across 4 independent experiments, 35 cells across 5 independent experiments and 31 cells across 4 independent experiments for control 1 µg/mL FN, control 50 µg/mL FN, PF-228 treated 1 µg/mL FN and PF-228 treated 50 µg/mL FN respectively. * denotes $p<0.05$; *** denotes $p<0.001$; # denotes $p<0.0001$.

**Figure S3-** Cross section through the duodenum of a mouse small intestine stained with phalloidin (cyan) and DRAQ5 (magenta) with a cell dividing near the base of the crypt, imaged using confocal microscopy. Magnified view corresponding to the dotted yellow rectangle is shown to the right.

**Figure S4**- A) XY views of HeLa cells at anaphase (top row) and metaphase (bottom row) stained for DAPI, microtubules (MT) and cenexin. Cenexin marks the mother centrosome, marked by dotted yellow circle. The daughter centrosome is marked by a dotted white circle. B) XZ views of the cells shown in (A) showing microtubules and cenexin. C) Quantification of the proportion of cells with tilted spindles where the mother centrosome is preferentially tilted towards the substrate. Measurements were made for 15 metaphase spindles and 17 anaphase spindles across 3 independent experiments for each. Error bars represent SEM. Scale bar represents 5 µm.

**Supplemental Video Legend**

**Movie S1: TIRF time-lapse of EGFP-paxillin during metaphase through telophase**

Movie shows a HeLa cell expressing EGFP-Paxillin to mark adhesions undergoing mitosis starting from metaphase progressing through telophase on a high adhesive substrate.
Movie Length- 47.5 min. Horizontal and vertical dimensions, 73.5 µm and 55.4 µm, respectively.

## Figure S1- Related to Figure 1

A The contractile ring is more circular on the low FN

XZ views of contractile ring (yellow)

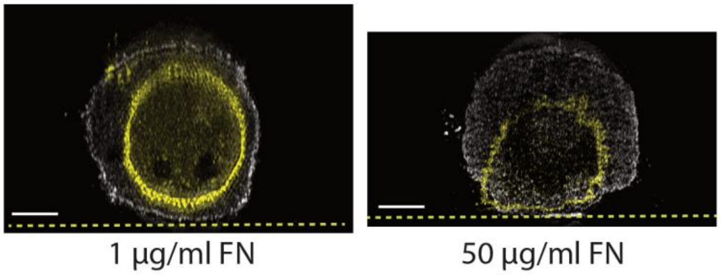


B Ring Aspect Ratio

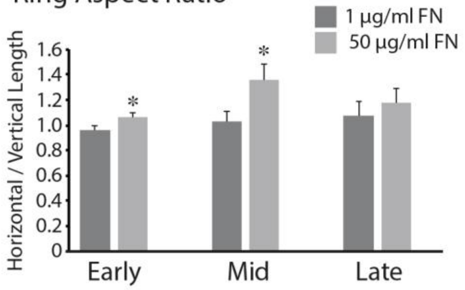


C Localization of endogeous paxiliin during furrow ingression

XY Views 1 µg/ml FN

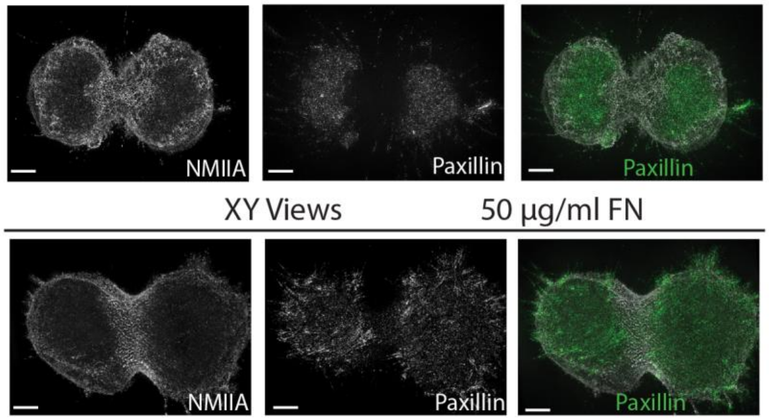

Figure S2- Related to Figure 2

A Western blots showing acute treatment with FAK inhibitor results in reduced pFAK levels

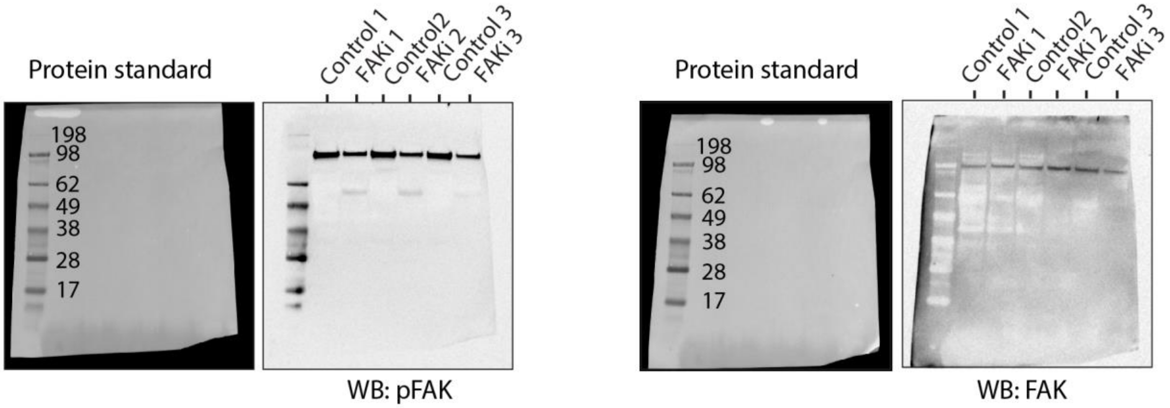


B Western blots validating knockdown of vinculin using siRNA

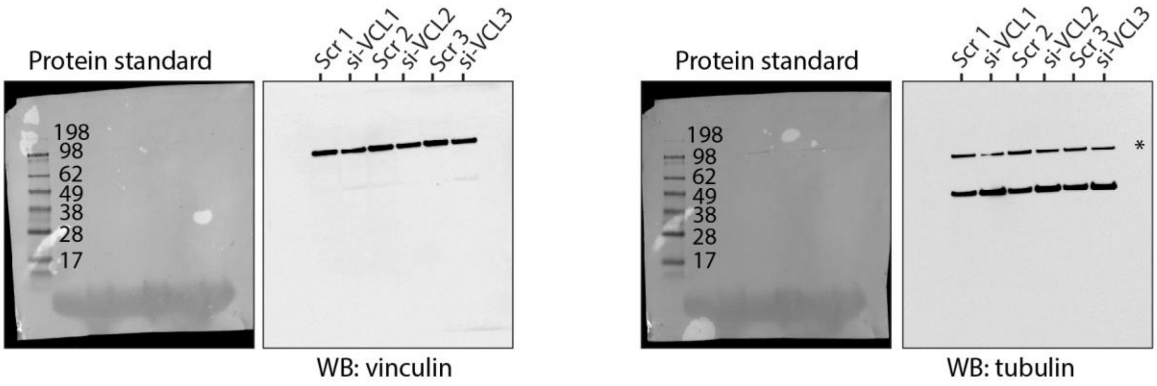


Spread area and paxillin intensity is greater on high FN and further increased upon PF-228 treatment

C Quantification of paxillin intensity in control and PF-228 treated cells

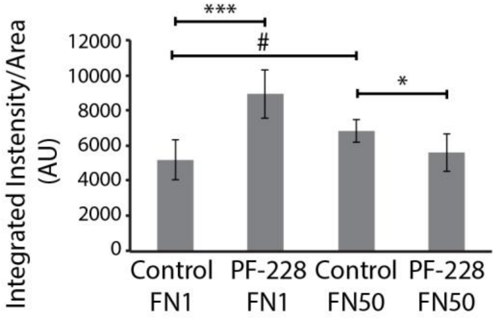


D Quantification of spread area in control and PF-228 treated cells

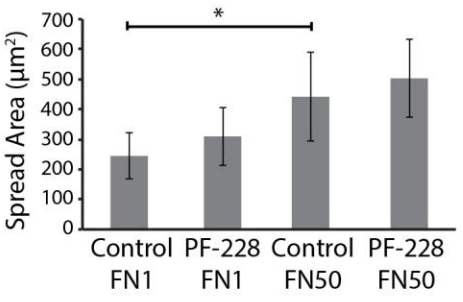

Figure S3- Related to Figure 3

Ingression proceeds from the basolateral domain in cells dividing in small intestinal crypts

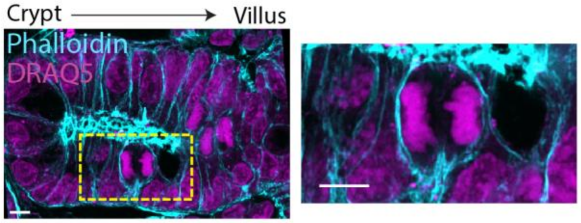

Figure S4 - Related to Figure 4

The mother centriole is preferentially tilted towards the substrate during metaphase and anaphase

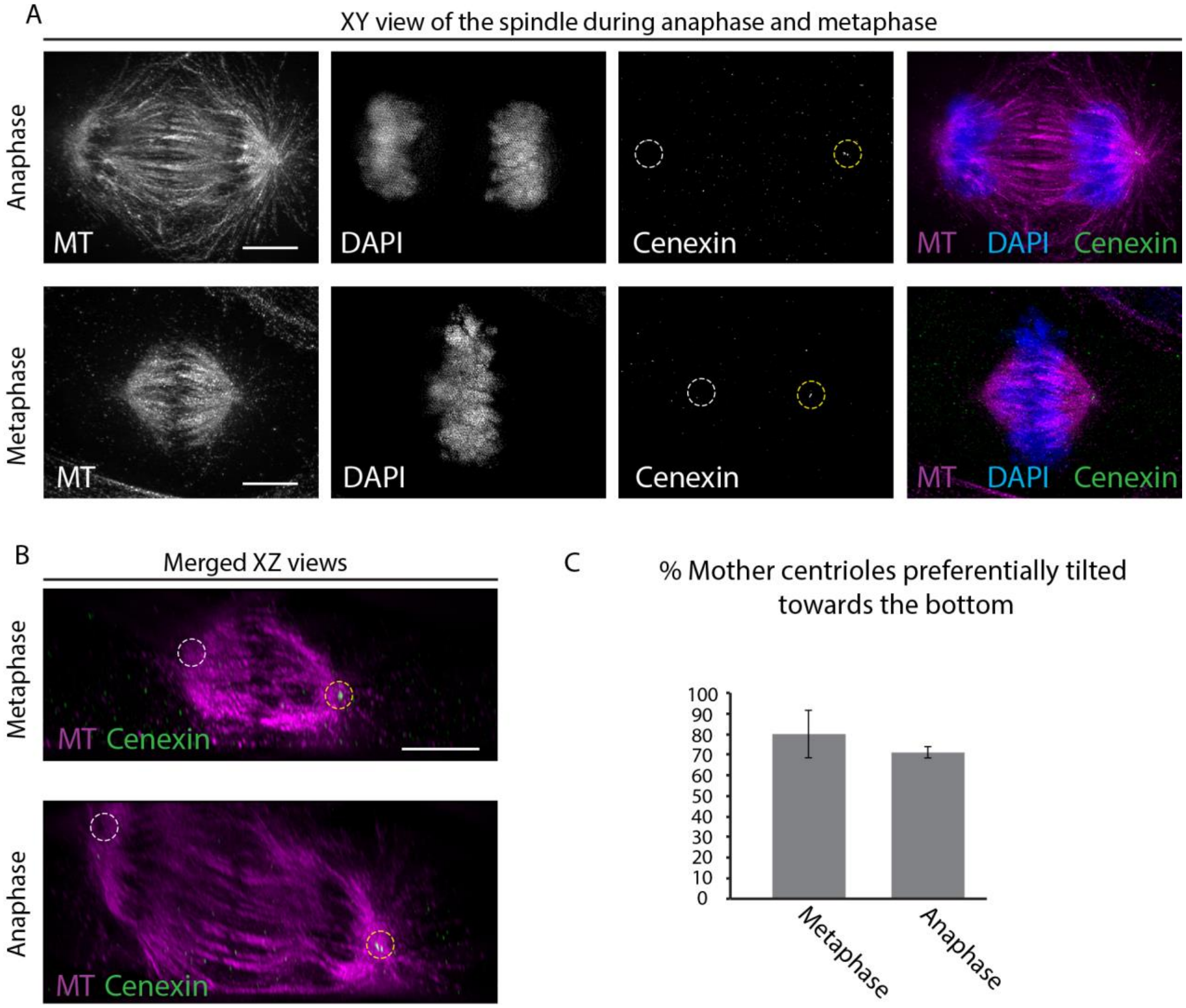

Supplement: Supplementary Information [file srep29846-s1.pdf]
